# Identification of human short introns

**DOI:** 10.1371/journal.pone.0175393

**Published:** 2017-05-17

**Authors:** Emmanuel L. Abebrese, Syed H. Ali, Zachary R. Arnold, Victoria M. Andrews, Katharine Armstrong, Lindsay Burns, Hannah R. Crowder, R. Thomas Day, Daniel G. Hsu, Katherine Jarrell, Grace Lee, Yi Luo, Daphine Mugayo, Zain Raza, Kyle Friend

**Affiliations:** Department of Chemistry and Biochemistry, Washington and Lee University, Lexington, Virginia, United States of America; International Centre for Genetic Engineering and Biotechnology, ITALY

## Abstract

Canonical pre-mRNA splicing requires snRNPs and associated splicing factors to excise conserved intronic sequences, with a minimum intron length required for efficient splicing. Non-canonical splicing–intron excision without the spliceosome–has been documented; most notably, some tRNAs and the *XBP1* mRNA contain short introns that are not removed by the spliceosome. There have been some efforts to identify additional short introns, but little is known about how many short introns are processed from mRNAs. Here, we report an approach to identify RNA short introns from RNA-Seq data, discriminating against small genomic deletions. We identify hundreds of short introns conserved among multiple human cell lines. These short introns are often alternatively spliced and are found in a variety of RNAs–both mRNAs and lncRNAs. Short intron splicing efficiency is increased by secondary structure, and we detect both canonical and non-canonical short introns. In many cases, splicing of these short introns from mRNAs is predicted to alter the reading frame and change protein output. Our findings imply that standard gene prediction models which often assume a lower limit for intron size fail to predict short introns effectively. We conclude that short introns are abundant in the human transcriptome, and short intron splicing represents an added layer to mRNA regulation.

## Introduction

Most pre-mRNA introns are excised by the major or minor spliceosome (reviewed in refs[1,2]). Both spliceosomes excise introns via similar reaction mechanisms, leading to exon-exon ligation. For both spliceosomes, intron removal requires conserved intronic sequence elements at the 5' and 3' splice sites as well as at the intronic branchpoint [3–8]. Conserved sequences differ; for major-class introns, the 5' and 3' ends of the intron contain conserved GU-AG nucleotide sequences [3–5]. In contrast, the minor-class spliceosome often uses conserved intronic AU-AC nucleotide sequences at the 5' and 3' ends [7,8]. In addition, the intronic branchpoint interacts with either major-class U2 snRNA (small nuclear RNA) or minor-class U12 snRNA during splicing, and other sequences such as the polypyrimidine tract toward the intronic 3' end are critical for optimal splicing [9–11]. Combined, these various sequences create a minimum intron length required for efficient splicing with splicing efficiency decreasing as intron size is reduced [12,13]. Conserved intron ends and a lower size limit are commonly used during RNA-Seq analysis when identifying introns and predicting gene structure [14,15].

During RNA-Seq, introns are predicted most effectively by checking for conserved intron sequences. Introns are initially indicated when sequencing reads align discontinuously with the reference genome; the gaps in sequencing alignments, or putative introns, are then further investigated. If the sequence of the gap begins and ends with GU-AG, AU-AC, or closely-related sequences, and the length is greater than a user-defined minimum (often 50 nucleotides), then the intron is defined. The flanking sequences are considered exons [14,15]. These are important considerations when performing sequence alignments since spontaneous genomic deletions are common, especially in tissue culture lines [16,17]. Such deletions would frequently contaminate predicted intron pools if canonical intronic sequence constraints were ignored.

But non-canonical introns do exist. Some spliceosomal introns have degenerate splice site sequences. RNA-Seq alignment algorithms commonly deal with the most abundant degeneracy, GC-AG rather than GU-AG, but other splice site sequences are tolerated *in vivo* [7]. More importantly, not all introns are removed by one of the two spliceosomes. Some pre-tRNAs contain short introns removed by a specialized splicing machine, the splicing endonuclease (SEN) complex, which functions in the vertebrate nucleus [18] (yeast SEN complex is at the mitochondrial membrane [19]). In addition, one well-documented short mRNA intron is removed by a non-spliceosomal mechanism. Cellular stress activates the endonucleolytic activity of IRE1 (iron response element binding protein 1 [20–23]). IRE1 then cleaves target mRNAs that can be ligated back together, removing a short intron. In *S*. *cerevisiae*, tRNA ligase completes splicing of the *HAC1* (homologous to ATF/CREB 1) mRNA [21], and in humans, RtcB (RNA 3'-terminal phosphate cyclase) completes splicing of a separate mRNA which encodes XBP1 (X-box binding protein 1 [24]). In both cases, intron removal shifts the translational reading frame so that a new protein is expressed. In both yeast and mammals, IRE1 excises a short intron (29 and 26 bases respectively [20–22]). The introns removed by IRE1 and the SEN complex are short and found in structured RNAs, and it is unknown how many other short introns exist.

With RNA-Seq datasets, there is a wealth of sequencing data that can be used for short intron identification, but conventional sequencing analysis overlooks this intron class. Recently, one group has attempted to identify short introns [25,26]. They successfully identified canonical short introns under 50 bases in length and implicated the major-class spliceosome in short intron removal [25]. Another group identified a few short introns likely excised by IRE1 [27]. In each case, the goal was to identify introns spliced by known splicing machines, the spliceosome and IRE1 respectively. However, non-canonical introns could be spliced by an array of unidentified enzymes that were overlooked in these earlier studies.

Here, we have identified short introns more comprehensively. We report the identification of hundreds of human short introns that range in length from 10 to 70 bases. Many short introns are found in mRNA open-reading frames and would be expected to alter protein output when spliced. A majority of short introns maintains major-class splice site consensus sequences, but many do not. We identify these short introns not only in mRNAs, but also in long non-coding RNAs (lncRNAs). Short introns are enriched for secondary structure. As is true for the short intron in *XBP1* mRNA, newly-identified short introns are not constitutively spliced, but rather are alternatively spliced. Taken together, our findings extend the intron family to include hundreds of additional short introns.

## Results

### Scheme for short intron identification

Intron prediction algorithms commonly exclude any predicted intron less than 50 nucleotides in length [15]. Since both human pre-tRNA and the *XBP1* mRNA short introns are shorter than this cutoff, we extended the normal search parameters to include introns with a predicted length down to 10 nucleotides. We chose this lower limit for two reasons: genomic deletions are infrequent at lengths greater than 10 nucleotides [16,17], and known short introns are longer than 10 nucleotides [20–22,25–27]. We were still concerned that spontaneous genomic deletions would contaminate our predicted intron pool, so we designed additional criteria to remove these deletions from subsequent analysis.

**Fig 1:**
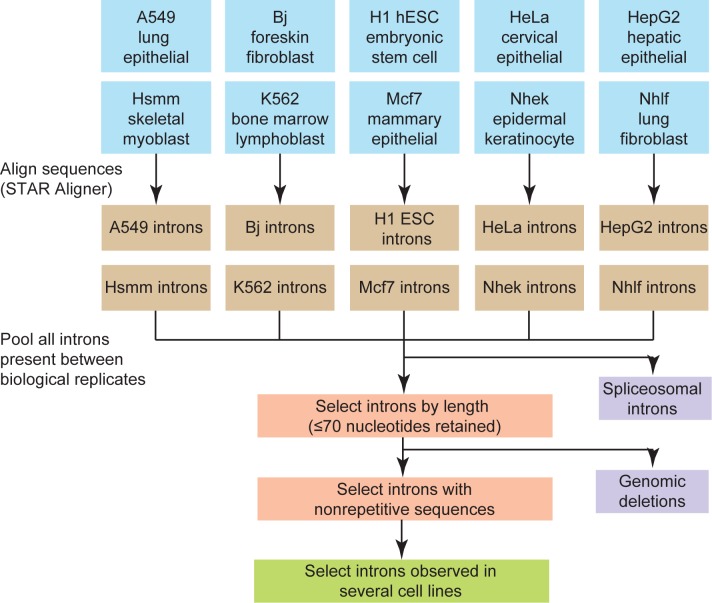
Summary of short intron identification strategy. Ten different cell lines, representing different tissue types, were analyzed. RNA-Seq data for two biological replicates for each cell line were aligned to the human genome using the STAR sequence aligner [29]. Predicted introns were compared between replicates and included if they were identified with more unique sequencing reads than an experimentally-determined threshold (see Fig 2D). Short introns were then selected based on size, and repetitive sequences were removed from the predicted intron pool. Predicted short introns were then compared to unannotated, longer introns on the basis of conservation between cell lines to finalize an intron pool.

We provide an overview for our workflow to identify short introns (Fig 1).We first selected ten RNA-Seq datasets deposited as part of the ENCODE project [28]. Each dataset corresponds to a different human cell line with two biological replicates. Importantly, these datasets were generated using the same methodology making downstream comparison between cell lines possible. Replicate datasets were used to screen for reproducibility after sequence alignment. We aligned the replicate RNA-Seq datasets for each human cell line using the STAR sequence aligner [29] and the human reference genome. The STAR aligner was used since it can identify very short introns and includes the option to search for non-canonical splice sites, *i*.*e*. splice sites that lack either GU-AG or AU-AC consensus sequences. STAR alignment produced many predicted short introns which we filtered extensively to remove predicted introns with little experimental support as well as likely genomic deletions. Briefly, we used between-replicate reproducibility, removed likely genomic deletions, and checked for conservation in multiple cell lines (Fig 1). These latter steps are detailed in later sections.

### Non-canonical introns are predicted primarily at lengths below 70 nucleotides

**Fig 2:**
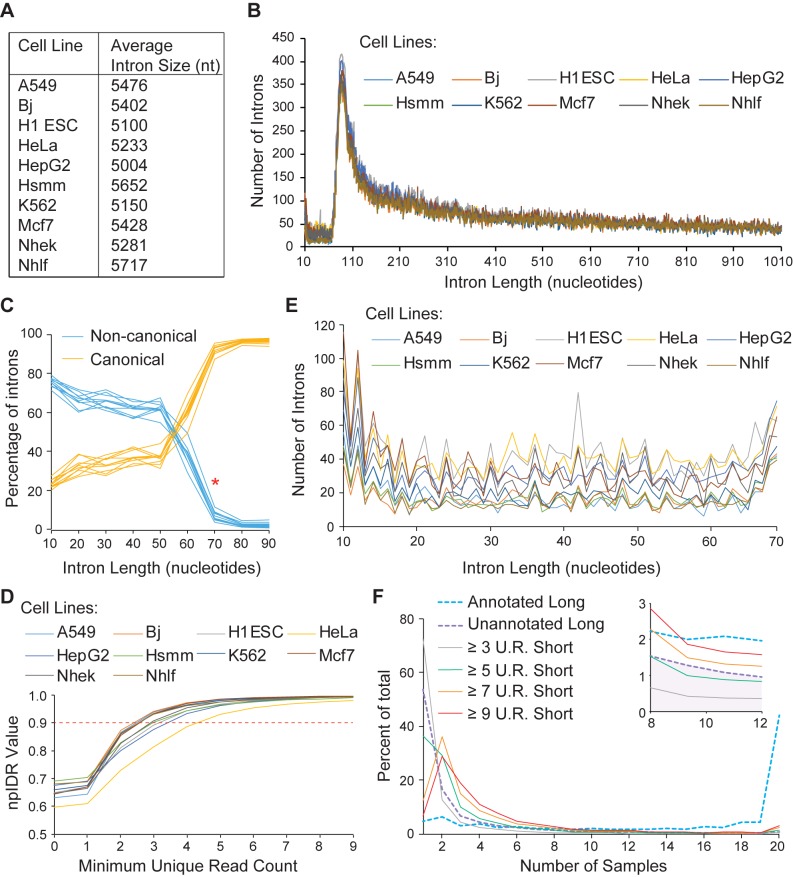
A pool of potential introns was extensively screened for high-quality short introns. (A) For each cell line, average intron length was calculated, ranging between 5 kb and 6 kb, consistent with the work of others [30,31]. (B) For each cell line, introns were binned and counted according to length (10 nt to 1010 nt are shown). The data are consistent between cell lines, and in each case, there is a peak at ~86 nt. (C) Canonical introns (GU-AG, GC-AG, and AU-AC for major or minor class spliceosomes) were separated from non-canonical introns. We calculated the percentage of introns that were either canonical or non-canonical over a range of sizes (from 10 nt to 90 nt) for each cell line (shown as multiple lines on the graph). At 70 nt (*), there is a sharp decrease in the percentage of non-canonical introns. (D) A non-parametric Irreproducible Discovery Rate (npIDR) was calculated for short introns between each pair of biological replicates. We repeated the calculation with increasing numbers of minimum sequencing read support for each cell line. To be included in subsequent analysis, predicted intron pools had to cross an npIDR = 0.90 threshold, meaning that introns in the final pool were consistent 90% of the time between biological replicates. (E) Remaining introns were then binned according to length as in (B) and counted over the range from 10 nt to 70 nt. The remaining predicted intron pool was fairly evenly distributed by size, but with a peak at 10 nt. (F) After removing likely genomic deletions, we compared our remaining short intron pool to both annotated and unannotated longer introns. Annotated longer introns were present in the largest number of samples, whereas unannotated longer introns were present in considerably fewer samples. We then checked for short intron conservation across samples as a function of increasing read support (≥ n U.R. Short; where n is the number of unique reads). With more read support, short introns were found in a larger number of biological samples. Note: inner window is a zoomed in view of the region between 8 and 12 on the x-axis.

Sequencing alignments returned many predicted introns (~300,000 per sample) which we first compared to other published intron datasets. First, we assessed the average lengths for all predicted introns to ensure that our search parameters returned results consistent with the work of others. Fig 2A contains a table with values for the average predicted intron length separated by biological sample. These average values are consistent between cell lines and are also consistent with previously-reported, average human intron size [30]. We next focused on introns that were less than ~1000 nts in length. Plotted in Fig 2B is a count of the total number of introns at various intron lengths. These profiles are separated by cell line, and for all samples there is a peak at ~86 nts (Fig 2B). These data indicate that the parameters used in our current sequencing alignments returned results generally consistent with the work of others [31,32] and consistent between samples.

Next, we focused on identifying short introns. Since the splicing machinery has been documented to require a minimum intron length [12,13], we queried predicted introns for canonical (GU-AG, GC-AG, or AU-AC) or non-canonical ends. We then binned these data according to intron length; the data are plotted in Fig 2C. At progressively shorter intron lengths, predicted introns favor non-canonical ends. At lengths greater than 70 nts, the majority of introns have canonical ends, and these predicted introns are likely spliced by either the major or minor spliceosome. Note that the number of canonical introns at shorter lengths is higher than expected by chance (~20% versus 1.2% expected by chance); this number may be inflated by the STAR alignment algorithm which is biased to detect canonical introns. Since non-canonical introns predominate at sizes <70 nts, we used this length as an upper bound in subsequent analysis.

### Additional filtering was used to assign short introns

At this point, many predicted short introns could have been observed due to spontaneous genomic deletion in the various cell lines, and some additional short introns, in many cases, had little sequencing read support. We therefore further filtered the predicted short intron sequences.

For each cell line, the ENCODE data contained two biological replicates making it possible to do pairwise comparisons. Normally, a false discovery rate could be calculated comparing predicted introns with a reference set, but there is no reference set for short introns. Therefore, we employed the non-parametric Irreproducible Discovery Rate (npIDR) approach [33,34]. With npIDR, predicted introns were first binned according to the number of unique sequencing reads that supported the intron. The presence/absence of each intron was then compared between biological replicates (Fig 2D) with values closer to 1 indicating a higher degree of conservation between samples. As expected, with greater sequencing read support, introns were more likely to be present in both biological replicates. These analyses are biased toward more abundant transcripts, so greater sequencing depth could facilitate the identification of more putative introns. Here, our desire was to ensure reproducibility, so we used an npIDR cutoff of 0.90, meaning that each remaining intron was present in a population conserved 90% of the time between biological replicates, although not necessarily between cell lines.

We next attempted to remove spontaneous genomic deletions from our predicted intron pool. Tissue culture cell lines contain various genomic abnormalities, including aneuploidies, genomic insertions, and genomic deletions [16,17]. Of these, spontaneous genomic deletions can give rise to sequencing reads that predict novel exon-exon junctions since a gap is generated when the sequencing read is aligned to the reference genome. We first questioned whether our pool of predicted introns contained any genomic deletions since longer (≥10 nucleotide) deletions are rare [16,17]. We repeated the analysis performed in Fig 2B, but with a focus on the region of interest (predicted intron lengths between 10 and 70 nts). These data are shown in Fig 2E, and there is a counterintuitive peak at very short intron lengths (10–12 nts in length) which suggested that spontaneous deletions still contaminated our predicted intron pool since known short introns are longer than 10–12 nucleotides [18–22]. Our next step was to adopt a sequence-based approach to eliminate genomic deletions from our predicted intron pool.

Spontaneous genomic deletions are common when repetitive DNA sequences are replicated by DNA polymerase [35]. For example, repeating mono-, di-, or trinucleotide sequences can give rise to higher rates of genomic deletion [36]. Genomic deletions also commonly occur when unwound DNA forms a secondary structure such as a stem-loop [37]. Therefore, we analyzed both predicted intron sequences as well as the flanking exons for repetitive sequences. Full results are contained in S1 Table. To summarize, 27.8% of the predicted “introns” are found in repetitive DNA. This contrasts with the ~17% of the human genome that contains repetitive DNA sequences at a size comparable to our predicted intron pool [38]. The majority of these likely genomic deletions arise in regions of the DNA that can form secondary structure (20%). We removed all likely genomic deletions from further analysis, leaving 10,417 predicted introns.

Many of the predicted short introns remaining in our pool were poorly conserved between cell lines. To further narrow down the number of predicted short introns, we took advantage of the fact that the search algorithm predicted many longer introns that were unannotated in the reference genome. We therefore compared these longer, unannotated introns against our pool of short introns. For each longer intron, both annotated and unannotated, we first used the npIDR cutoffs established above to remove predicted introns with little sequencing support. We then queried the number of samples (out of 20 total) containing the remaining longer introns. Results were tabulated and converted to a percentage of the total (shown in Fig 2F, separated by annotation). We then overlaid the same information for our predicted short intron pool with increasingly stringent cutoffs for the minimum number of unique reads supporting the intron (Fig 2F). Once each intron in the short intron pool had at least 7 unique sequencing reads to support it, the distribution of short introns favored detection in more biological samples than the unannotated longer introns. Using 7 unique reads as a cutoff, 3,027 predicted short introns remained. In summary, we extensively filtered predicted short introns to generate a high-quality pool of short introns.

### Short introns are alternatively spliced

**Fig 3:**
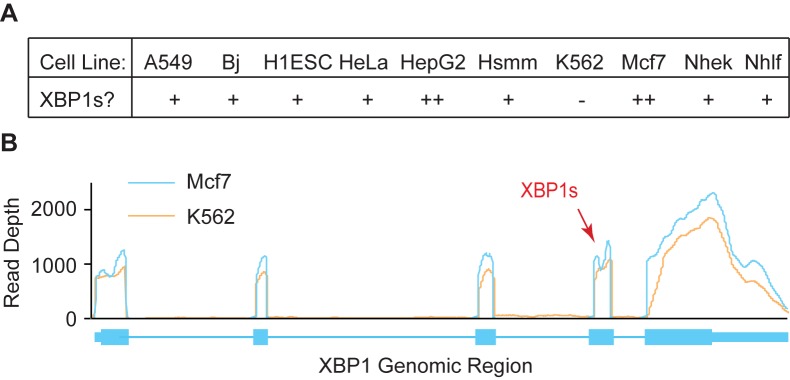
Summary of RNA-Seq analysis for the *XBP1s* intron. (A) Summarized are whether sequencing reads predicted the *XBP1s* intron in the cell lines listed. Sequence support was obtained in no (-), one (+), or two (++) samples as indicated. (B) For the Mcf7 and K562 cell lines, the sequencing read depth is plotted for the *XBP1* locus. The region where the *XBP1* mRNA short intron is found is indicated (XBP1s). Note the dip in sequencing read depth in this region only in the Mcf7 cell line, indicating some *XBP1s* splicing.

For mRNAs, the short intron in the *XBP1* mRNA (*XBP1s*) has been analyzed for splicing efficiency [22,39]; here, splicing efficiency varies depending on cellular growth conditions. When cells are growing normally, the short intron is spliced infrequently (~10% of the time), but when cells are stressed to induce the unfolded protein response, *XBP1s* splicing approaches 80–90% [22,39]. The ten cell lines used for our analysis were cultured under normal growth conditions, so we anticipated that *XBP1s* would be spliced inefficiently. In all but one cell line, we detected sequencing reads for exon-exon junctions resulting from *XBP1s* splicing (Fig 3A). We observed that *XBP1s* was spliced 3.7% of the time on average, with a range from 18.4% (in the Mcf7 cell line) to 0% (in the K562 cell line). When aligned sequencing reads were plotted versus genomic coordinates for the Mcf7 cell line, we observed a dip in sequencing reads at the position of *XBP1s* (Fig 3B). As for *XBP1s*, we anticipated that many other short introns might be spliced inefficiently, but we also considered the possibility that some short introns would be constitutively spliced.

**Fig 4:**
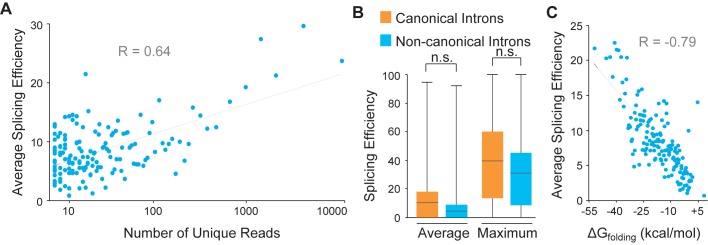
Short introns are both alternatively spliced and structured. (A) Short intron splicing efficiency was calculated for 3,027 predicted short introns. The introns were binned in groups of 20 according to sequencing read support, and splicing efficiency was plotted. There is a positive correlation between sequencing read support and splicing efficiency (R = 0.64, p < 0.01). (B) Short introns were separated according to whether they had canonical or non-canonical sequences at the ends of the introns. Both average and maximal splicing efficiency (across all cell lines) were calculated for every intron. Plotted are the box-and-whisker plots with the highest, lowest, 25^th^, 50^th^, and 75^th^ percentiles indicated. There was no significant (n.s.) difference between canonical and non-canonical intron splicing efficiency. (C) The free energy of folding (ΔG_folding_) was calculated for every short intron. The introns were ranked by folding energy and binned in groups of 20; average splicing efficiency per group was then calculated. The data are plotted showing a strong negative correlation between folding free energy and splicing efficiency (R = -0.79, p < 0.01).

To calculate splicing efficiency for the entire pool of predicted introns, we counted and compared the reads aligning to the intron interior versus the flanking exons. We analyzed our entire short intron pool in every cell line (even though most were not detected in every cell line). The complete results of our analyses are contained in S2 Table. We calculated average splicing efficiency, and the data are plotted in Fig 4A with the introns binned (in clusters of 20) according to the amount of sequencing read support. Most short introns were spliced inefficiently, but those introns that were spliced most efficiently, in general, had the most sequencing read support. These data suggest that short introns are typically alternatively spliced.

Since each short intron was alternatively spliced in at least one cell line, we next queried features of the short introns that might contribute to splicing efficiency. Surprisingly, splicing efficiency was not governed by whether the short intron contained canonical splice site sequences. Shown in Fig 4B is a chart of splicing efficiency for unbinned canonical introns versus unbinned non-canonical introns. Canonical introns were spliced slightly more efficiently, but the difference was not significant. However, the short introns with the greatest sequencing read support were predominantly canonical introns. For the top 100 short introns (based on sequencing read support), 85/100 contained canonical ends. Taken together, these data suggest that highly expressed short introns are predominantly canonical, but that splicing efficiency is not correlated with whether an intron is canonical or non-canonical.

The conserved sequences within a major-class intron recruit components of the splicing machinery, such as U1 snRNP and U2 snRNP [6,9]. During splicing, spliceosomal snRNPs associate with the intron ends and then loop out intervening intronic sequences. We hypothesized that short introns might facilitate splicing if the introns folded into structures with the 5ˈ and 3ˈ splice sites close in space. We used an RNA folding algorithm [40] to predict the thermal stability of every short intron in our pool. We ranked the introns according to folding free energies, clustered them in groups of 20, and calculated average splicing efficiencies. Shown in Fig 4C is the strong negative correlation between folding free energy and splicing efficiency; that is, more stable secondary structure predicts higher splicing efficiency. These data argue that short introns fold into rigid secondary structures in order to promote splicing.

### Short introns can be separated into different classes

To examine short introns in more detail, we selected the 600 short introns with the greatest sequencing support, ranging from 50 unique sequencing reads to 37,514 unique reads. These short introns fell into three main categories: canonical pre-mRNA introns, potentially spliced by the spliceosome; non-canonical pre-mRNA (or mRNA) introns; and short introns within lncRNAs (all data can be found in S3 Table). Among the 600 short introns, 578 were in regions of the genome with known transcripts; 225 short introns were annotated in the reference genome or had transcriptional support from ESTs. Thirty-four short introns were alternatively spliced using one known splice site with 29 of these using the annotated 5ˈ splice site, but a different 3ˈ splice site. Two hundred, fifty-one short introns were contained in UTRs with the majority (240 short introns) coming from 3ˈ UTRs. Ninety-eight unannotated short introns were removed from ORFs, and when spliced, 50 of these introns should frameshift the translational reading frame. Lastly, 39 pre-mRNAs were identified with multiple short introns within this reduced dataset. We discuss each group of introns: canonical, non-canonical, and lncRNA introns in depth below.

### Canonical short introns may be spliced unconventionally

**Fig 5:**
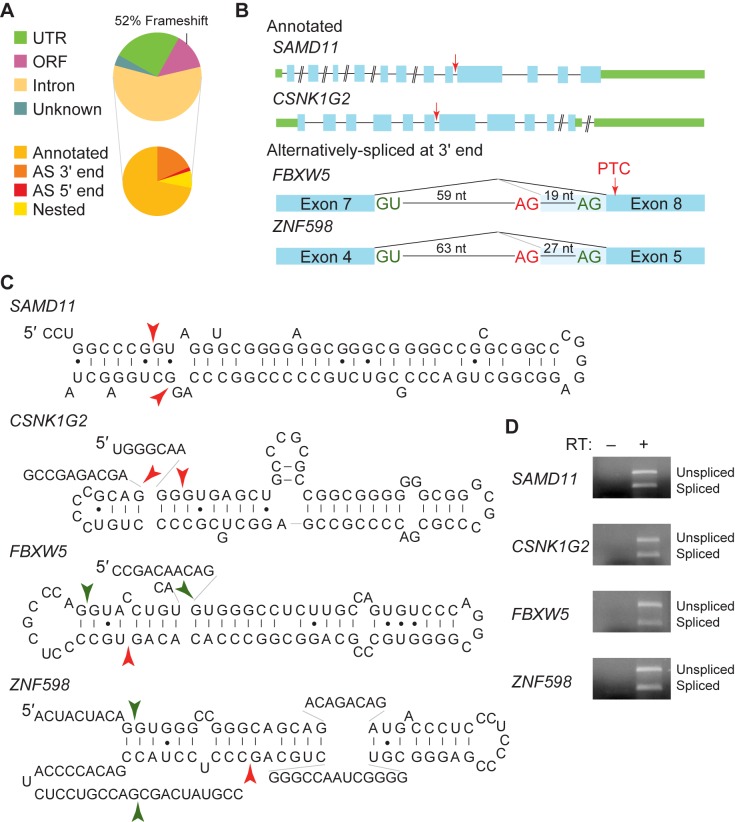
Canonical short introns may be produced by unconventional splicing. (A) Of the 600 short introns with the greatest sequencing read support, canonical introns were examined for their positions within mRNAs. The majority were known introns, with annotated 5ˈ and 3ˈ splice sites (Annotated), but a smaller number were unannotated, using a known splice site and an unannotated 3ˈ (AS 3ˈ end) or 5ˈ (AS 5ˈ end) splice site for intron excision. For short introns in ORFs, 52% of these are predicted to change the mRNA reading frame. (B) A small subset of short introns is examined more closely. Shown are the *SAMD11*, *CSNK1G2*, *FBXW5*, and *ZNF598* loci. The *SAMD11* and *CSNK1G2* loci both encode pre-mRNAs harboring annotated short introns. Note that both loci encode pre-mRNAs with clusters of short introns (with the shortest intron indicated by a red arrow). The *FBXW5* and *ZNF598* loci both encode pre-mRNAs that are likely alternatively spliced to yield a short intron. In both cases, the flanking exons and intron are shown. For the *FBXW5* mRNA, it is predicted that short intron splicing would change the reading frame of the protein product resulting in a premature termination codon (PTC). (C) For *SAMD11*, *CSNK1G2*, *FBXW5*, and *ZNF598* pre-mRNAs, the short intron with ten nucleotides from each flanking exon was folded computationally. Note that each intron folds into extensive secondary structure with intron ends positioned in proximity. Intron ends are indicated with arrowheads, and for *FBXW5* and *ZNF598* pre-mRNAs, both the annotated splice sites (green) and alternate splice sites (red) are indicated. (D) We used RT-PCR to confirm short intron removal in the *SAMD11*, *CSNK1G2*, *FBXW5*, and *ZNF598* pre-mRNAs. For the *FBXW5* and *ZNF598* pre-mRNAs, primers were designed to specifically detect short intron splicing (one primer sequence within the alternatively spliced region); for the other pre-mRNAs, primers were located outside the short intron to detect both pre-mRNA and spliced mRNA. As predicted from our splicing efficiency analysis, every short intron was alternatively spliced. Samples that omitted reverse transcriptase (from the RT step) serve as a control for contaminating DNA.

A slight majority (322) of the short introns with the most read support are canonical introns–those with GU-AG, GC-AG, or AU-AC sequences at the ends. The majority (319/322) contain the major-class splice site sequences, rather than AU-AC, arguing that these introns may be removed by the major-class spliceosome, although it should be noted that the minor spliceosome can remove introns with major-class splice sites [2,41]. We first asked which regions of a pre-mRNA contain canonical short introns. Shown in Fig 5A are canonical short intron locations: in pre-mRNA UTRs, introns, or ORFs (meaning coding exons). Most canonical introns overlapped with established pre-mRNA introns, and 71.8% of these used established 5ˈ and 3ˈ splice sites (Fig 5A). The remaining, unannotated introns largely use an annotated 5ˈ splice site, but an alternate 3ˈ splice site. Very few (8.0%) used neither a known 5ˈ nor 3ˈ splice site (Nested, in Fig 5A). For the alternatively spliced introns, the alternate splice site was typically within 50 nt of an annotated splice site (in 34/35 instances). Since in many cases, known splice sites were used in canonical short intron excision, these data implicate a spliceosome in canonical short intron removal.

We further examined a few pre-mRNAs in more detail to understand canonical short intron removal. Shown in Fig 5B are four loci: the *SAMD11*, *CSNK1G2*, *FBXW5*, and *ZNF598* loci, all of which contain canonical short introns. The *SAMD11* and *CSNK1G2* pre-mRNAs have annotated short introns, and these short introns are contained in pre-mRNAs with multiple smaller introns (Fig 5B). Since short introns likely have extensive secondary structure, we predicted the structure of *SAMD11* and *CSNK1G2* introns (with 10 nt from the flanking exons); the most energetically-preferred structures are shown in Fig 5C. Strikingly, both pre-mRNAs are expected to fold into extensive hairpin structures that position the 5ˈ and 3ˈ splice sites close in space. In accordance with the splicing efficiency analysis, we observed that both introns were alternatively spliced (Fig 5D). We also examined the *FBXW5* and *ZNF598* pre-mRNAs which are alternatively spliced (Fig 5B). Interestingly, both pre-mRNAs contain other small annotated introns (78 nts for *FBXW5* and 90 nts for *ZNF598*). For *FBXW5*, usage of the more proximal 3ˈ splice site would result in a truncated protein. When the *FBXW5* and *ZNF598* pre-mRNA introns are folded (including both 3ˈ splice sites), both the annotated and unannotated 3ˈ splice sites are predicted to be close in space to the annotated 5ˈ splice site (Fig 5C). As above, we were able to confirm that both introns were alternatively spliced (Fig 5D). An intriguing possibility is that splicing could be regulated by preventing usage of one or the other 3ˈ splice site, potentially by docking of an RNA-binding protein.

### Non-canonical short introns likely include additional IRE1 targets

**Fig 6:**
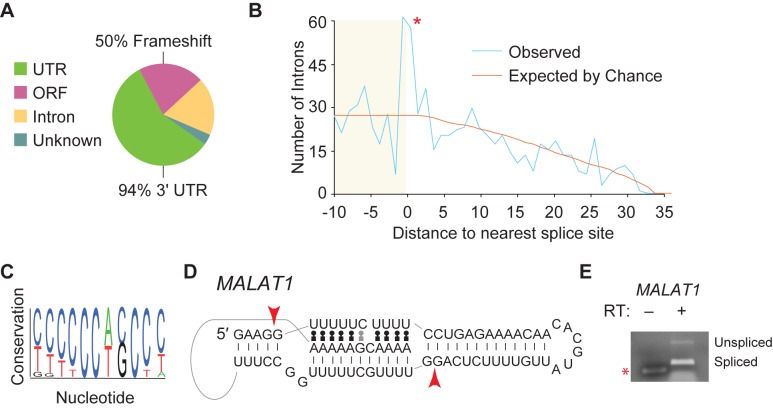
Non-canonical introns may include additional IRE1 targets, and lncRNAs can harbor short introns. (A) Non-canonical introns were analyzed as in Fig 5A. Here, however, the majority of introns were found in mRNA 3ˈ UTRs. Of those short introns that lay in ORFs, 50% are predicted to change the translational reading frame. (B) Short introns were screened for the IRE1 consensus sequence that has been identified from both *XBP1* mRNA and RIDD targets [44]. Of the 861 mRNAs with the consensus sequence, we calculated the distance between the consensus sequence start and the exon/intron boundary (0 on the x-axis). There is a striking and significant (p < 0.01) peak at the exon/intron boundary indicating that many of these introns were processed at an IRE1 consensus sequence. (C) MEME analysis was done for the non-canonical introns, and one significant (p < 0.01) motif was returned (shown). The motif is very cytidine-rich. (D) One of the four short introns from the *MALAT1* lncRNA is shown. The splice sites (arrowheads) are located proximal to gaps in secondary structure. Note that intron excision removes sequences that are capable of forming a triple helix in *MALAT1*. (E) We used RT-PCR to confirm short intron removal from *MALAT1* lncRNA. Primers were designed to detect both pre-mRNA as well as spliced mRNA. As predicted from our splicing efficiency analysis, the short intron was alternatively spliced, although the spliced form predominates. The sample that omitted reverse transcriptase (from the RT step) serves as a control for contaminating DNA with a shorter product observed (*).

Most of the short introns with the most read support are canonical, but many short introns (255 total introns) lack splice site consensus sequences for either the major or minor spliceosome. A few non-canonical introns (43 total) are annotated based on EST data, but the remaining 212 non-canonical introns are unannotated (S3 Table). Most of the non-canonical introns were identified in mRNAs (246/255 total), and 58 of these short introns were contained in mRNA coding regions (Fig 6A). Intron excision is predicted to alter translational reading frame for 29/58 of the short introns present in coding regions. The majority of non-canonical introns were present in 3ˈ UTRs (160/255). Spliceosomal introns are rarely found in 3ˈ UTRs since an intron downstream of a normal stop codon can induce nonsense-mediated mRNA decay (NMD [42]). An interesting possibility is that short introns may be present in 3ˈ UTRs to bypass NMD while allowing alternative splicing. A full list of non-canonical introns can be found in S3 Table.

The *XBP1s* intron is a non-canonical short intron excised by the RtcB/IRE1 complex [22,24]. We queried our reduced intron pool for mRNAs that are involved in the unfolded protein response and for factors that are known targets of RIDD (IRE1-dependent decay) since both involve IRE1 activity [43]. We identified *XBP1* as well as *ATF4*, *CES1*, *GYLTL1B*, and *RTN4* mRNAs as containing short introns identified in our study. Since there are 21 known IRE1 target mRNAs [44], we have identified about a quarter of them here. Importantly, these data suggest some overlap between RIDD and IRE1-mediated splicing since *CES1*, *GYLTL1B*, and *RTN4* are all known RIDD targets.

We next asked whether our short intron pool was enriched for the IRE1-mediated mRNA cleavage consensus: CTGCAG [44,45]. In addition to the consensus, we also queried known variants. By random chance, we expected to identify 202 occurrences of IRE1 target sites, but we found 861 occurrences meaning that the IRE1 cleavage site was significantly enriched in our short intron pool (p<0.01). We clustered the introns that contained potential IRE1 cleavage sites, and tested how far the cleavage site was from the exon/intron boundary. There was significant enrichment at or within 1 nt of the exon/intron junction (Fig 6B) suggesting that the short intron pool contains many additional IRE1 target mRNAs.

Among the non-canonical introns there are potentially other factors involved in their excision. As a final step, we analyzed the short introns for additional conserved sequences using Multiple Em for Motif Elicitation (MEME [46]). Apart from the IRE1 consensus, the only sequence that was enriched is shown in Fig 6C (all introns with the consensus sequence are in S4 Table, arguing that many short introns lack an established motif. Taken together, these data suggest that many additional IRE1 targets are present in the non-canonical short intron pool as well as known targets. Other enzymatic activities likely play a role in non-canonical intron excision.

### A few short introns are present in lncRNAs

Finally, a very small number of short introns were identified in lncRNAs. This is unsurprising since short introns are generally spliced inefficiently, and lncRNAs are often low abundance transcripts compared to mRNAs [47]. That we identify lncRNA short introns suggests that our analysis does not completely omit lower abundance transcripts. Among the few lncRNAs that contain short introns (S3 Table), we identified four short introns in the *MALAT1* lncRNA, [48,49]. *MALAT1* contains an unusual 3' end that is formed when RNase P cleaves the transcript [50]. One of the identified short introns in *MALAT1* overlaps with the second U-rich motif [51,52] required to form a triple helix to stabilize the transcript (Fig 6D); intron removal could eliminate this region and destabilize *MALAT1*, promoting *MALAT1* degradation. However, we observed high levels of the spliced transcript relative to the unspliced transcript calling that model into question (Fig 6E). Most of the remaining lncRNA-associated short introns map to Polycomb-associated RNAs [53]. Short intron removal may serve some function in these lncRNAs, but at this time, that function is unclear. In summary, we observe short introns in lncRNAs in addition to mRNAs.

## Discussion

Here, we have designed an approach to identify short introns from RNA-Seq data. As a result of our analyses, we identify 3,027 putative short introns which fall into canonical and non-canonical categories. The canonical introns contain major-class splicing consensus sequences at intron ends whereas the non-canonical introns do not. These short introns are present in both coding mRNAs as well as lncRNAs. When present in coding mRNAs, short introns are often expected to alter protein output when excised. Short intron splicing efficiency seems to be governed largely by RNA folding; more thermally-stable introns are spliced more efficiently. These findings suggest a possible mechanism for how the spliceosome can remove short introns due to proximity between 5ˈ and 3ˈ splice sites in the folded RNA. For non-canonical introns, we identify many potential IRE1 targets even though the cells were cultured normally; despite these growth conditions, our analysis expands the potential repertoire of introns excised by this complex.

### Canonical short introns may be spliced by the spliceosome

Most of the canonical introns that we have identified in this study are longer than 50 nucleotides, a length below which splicing efficiency has been observed to decrease [12,13]. But a number of canonical introns (655 in total) are shorter. In addition, we observe a connection between short intron splicing efficiency and calculated intronic thermal stability suggesting that splicing is influenced by intron secondary structure, arguing that these introns may be removed by an unconventional mechanism. If true, how might these short introns be spliced? It is possible that these introns are not excised by the spliceosome at all, but by another factor that happens to recognize the same consensus sequences. We prefer a different model. The canonical introns identified here often use known splice sites (see Fig 5A). In addition, the work of others has indicated that short introns are excised in a spliceosome-dependent manner, although the evidence was indirect [25,26]. Here, we postulate that many short, canonical introns are excised by the spliceosome, but in an unconventional manner–that intron secondary structure can bring the 5ˈ and 3ˈ splice sites into proximity to promote splicing.

How would splicing in this scenario work? The answer may relate to circular RNA (circRNA) formation. Recently, there has been intense interest in circRNAs which are produced in an unconventional way by the splicing machinery. With circRNA formation, exons are ligated by backsplicing the 3ˈ splice site of an upstream exon to the 5ˈ splice site of a downstream exon. Further studies showed that extensive base-pairing in the “intron” that would be removed after splicing drove circRNA formation [54–56]. A similar mechanism could be at play here. The short introns in this study could make an excellent system to study the mechanism behind splicing sequences that use secondary structure to promote splicing.

### Non-canonical introns are likely spliced by diverse enzymes

Well-established non-canonical introns include introns in pre-tRNAs and in the *HAC1/XBP1* mRNAs that are spliced by the SEN complex and IRE1 (with tRNA ligase or RtcB respectively [18–24]). In these cases, processing occurs via endonucleolytic cleavage within stem loops. The facts that IRE1 consensus sequences and secondary structure are enriched in our pool of short introns suggest many potential IRE1 targets within our intron pool. Importantly, we find that many IRE1 sites lie at the exon/intron junction in our short intron pool.

These findings are true of a large number of non-canonical introns, but by no means all of them. One other motif was enriched in the intron pool (Fig 6C). Outstanding questions remain. How many enzymes are involved in splicing these non-canonical introns? What are the nucleases and ligases? Are any of the sequences identified here examples of self-splicing introns? For sequences with an IRE1 site, how many are, in fact, processed by IRE1? An interesting future research question will be to explore additional RNA endonuclease and ligase activities to see if they play an additional role in short intron excision. Another open question is where in the cell does splicing occur? Short intron removal is not restricted to the nucleus, and it is likely that many short non-canonical introns are spliced in the cytoplasm, as for the *XBP1s* intron [57–59]. Furthermore, what cellular signals or stresses may regulate splicing of these introns? Since the short introns identified in this study were almost always alternatively spliced, it seems likely that splicing may be regulated, or at least, affected by cellular growth conditions.

## Conclusions

Here, we expand the inventory of intronic sequences in the human genome to include many additional short introns. Many more RNA-Seq datasets exist, and our methodology can be extended to these additional datasets making further short intron identification probable. Our analysis includes human cell lines, and it will be interesting to identify tissue-specific short intron splicing as a possible means to alter protein expression. Since most short introns are alternatively spliced, how intron excision is regulated in response to cellular signaling events or in specific tissue environments is an outstanding line of future investigation. Our findings are a next step in understanding an underappreciated aspect of gene regulation, altered mRNA profiles due to short intron removal.

## Materials and methods

### High-throughput sequencing data and genome alignment

Human cell line RNA-Seq data were downloaded from the UCSC Genome Browser website: http://hgdownload.cse.ucsc.edu/goldenPath/hg19/encodeDCC/wgEncodeCshlLongRnaSeq/. Data were originated from the Cold Spring Harbor Lab as paired-end reads on an Illumina GA2x platform [28]. For the A549, Bj, H1 hESC, HeLa, HepG2, Hsmm, K562, Mcf7, Nhek, and Nhlf cell lines, the selected data were derived from whole cell, polyadenylated transcripts.

Genomic alignment was performed using the STAR alignment algorithm [29] with default parameters except: outSJfilterOverhangMin (20 12 12 12), alignIntronMin (10), and alignIntronMax (100000). Sequencing reads were aligned to the hg38 human genomic assembly. The alignments were performed at Washington and Lee on an Intel high performance computing cluster with 215 logical CPUs across 8 compute nodes.

Short introns were identified from the SJ.out.tab output files after STAR alignment. These STAR output files contain information on how many uniquely-aligned reads map across predicted exon-exon junctions. Biological replicates were used to calculate a non-parametric Irreproducibility Discovery Rate (npIDR) as described [34]. npIDR values were recalculated using progressively greater numbers of unique sequencing reads until npIDR > 0.90 for each biological replicate. Only those short introns with unique reads in excess of this lower limit were used for subsequent analysis. Then, intron lengths were calculated by subtracting the position of the 5' intron end from the 3' intron end, and only introns with a length <71 nucleotides were considered for subsequent analysis. For deletion screening and splicing efficiency calculations (see below), all predicted introns were aggregated.

### Genomic deletion screening

The next component of analysis was to analyze predicted introns for sequences that were likely to result in high-frequency genomic deletions such as repetitive sequences. BED files were created with coordinates encompassing ten nucleotides of the predicted 5' and 3' flanking exons as well as the predicted intron. Nucleotide sequences were obtained for these BED files using the Galaxy server and the hg38 genomic assembly.

For direct repeat analysis, if six nucleotides of the 5' flanking exon matched the final six nucleotides of the intron, or if the six nucleotides of the 3' flanking exon matched the first six nucleotides of the intron, the predicted intron was labeled a direct repeat and removed from subsequent analysis.

For inverted repeat analysis, the intron ends were considered. If the six nucleotides at the 5' end of the intron were complementary to the six nucleotides at the 3' end, the intron was labeled as an inverted repeat and removed from subsequent analysis.

For simple repeating sequence analysis, the intron sequences were considered. If an intron contained >75% of a single nucleotide (such as 8 A’s in a 10 nucleotide intron), it was removed from subsequent analysis. These criteria were then applied for any combination of di-, tri-, tetra-, and pentanucleotide repeats, *i*.*e*. if an intron was >75% repetitive sequence, it was removed from subsequent analysis.

In total, 14,425 predicted introns were analyzed, and 4,008 were removed due to these analyses (note that some predicted introns fell into multiple categories). The remaining 10,417 predicted introns were then compared to longer introns.

### Comparison with unannotated longer introns

All introns longer than 70 nts were pooled from the STAR sequencing alignments (as long as they contained the minimum number of unique reads as calculated from the npIDR analysis). Predicted intron ends were then compared to reference intron ends in the hg38 genome assembly to determine whether they were annotated or were unannotated. Introns were separated, and for every intron, total unique reads (summed from all samples) and sample number (the number of datasets with an intron) were calculated. Similar analysis was done for all short introns, omitting the annotation stage.

### Splicing efficiency analysis

As above, BED files were constructed for every short intron remaining in the pool. In each case, three BED files were generated, one with coordinates -15 and -5 from the intron 5' end, one with ten nucleotides centered on the middle of the intron, and one with ten nucleotides +5 and +15 from the intron 3' end. Then reads that aligned to each genomic interval were counted for every BAM alignment file (20 in total, two for each cell line) using the Galaxy server.

Splicing efficiency was calculated based on the reads aligning to the intron divided by the average of the reads aligning to either the 5' or 3' flanking exon; this value was then subtracted from 100. All negative splicing efficiencies were set to zero. Both average and maximum (across all 20 datasets) splicing efficiencies were calculated for every intron.

For RT-PCR experiments, it was necessary to use a combination of human cell lines, the A549 and HepG2 cell lines. These were cultured under standard conditions: for the A549 cell line (F-12K medium, 10% FBS, and penicillin/streptomycin) and for the HepG2 cell line (EMEM, 10% FBS, and penicillin/streptomycin). Whole cell RNAs were isolated using TRIzol Reagent according to the manufacturer’s instructions (ThermoFisher Scientific). Reverse transcription was performed using random nonamers for priming and M-MuLV reverse transcriptase, according to the manufacturer’s instructions (New England Biolabs). Reverse transcription reactions derived from the HepG2 cell line were used for PCR amplification of the *CSNK1G2*, *FBXW5*, and *ZNF598* mRNAs. Those from the A549 cell line were used to amplify the *SAMD11* and *MALAT1* RNAs. Primer sequences are: *SAMD11* (GGAGATGTTCGCCTGGCAGC and CGTGGTTCAGCACCAGCAGG), *CSNK1G2* (AGCAGAGCCGCCACGAC and CTGGGAAGTTCTCGCAGAGC), *FBXW5* (TGGCAGGATCTGCTTGATGC and GGCAGACAGCAAGCAAGTCC), *ZNF598* (TGGCAGGAGATGGGGTGTCG and GAGCTGCTTAAGCACCTGCG), and *MALAT1* (GGCCAAGCTAGCATCTTAGC and TCCTGGAAACCAGGAGTGCC). PCR products were visualized on an agarose gel.

### Average splicing efficiency versus unique reads and folding energies

For the data in Fig 4A (comparing unique reads to splicing efficiencies), the 3,027 short introns were ranked according to the pooled (from all datasets) number of unique reads. The data were binned into 151 groups of 20 short introns, with progressively less unique reads in each subsequent bin. Within each bin, the average number of unique reads and average splicing efficiencies were calculated.

To calculate folding energies, every short intron sequence (as well as 10 nt from both the 5ˈ and 3ˈ flanking exons) were analyzed using the Mfold algorithm (RNA Quikfold at unafold.rna.albany.edu). In each case, the minimum free energy was taken for subsequent analysis. Introns were ranked according to free energies and binned in groups of 20 introns, with progressively lower free energies in each subsequent bin. Within each bin, both thermal energies and splicing efficiencies were averaged.

### Non-canonical intron analysis

The search for potential IRE1 sites was done on all 3,027 short introns (with 10 nt from both flanking exons). Five sequences were sought: CTGCAG, CCGCAG, CAGCAG, CTGCCG, and CTGCAA since these are known IRE1 cleavage sites [44,45]. 861 short introns contained these sites; note that many had multiple sites. The 5ˈ position for each sequence was identified, and the distances to both exon/intron boundaries were calculated. The minimum was taken to indicate which exon/intron boundary was closer.

Short non-canonical introns were also subjected to MEME analysis [46]. MEME was performed (meme-suite.org) on all non-canonical introns with a minimum motif size of 6 and a maximum motif size of 10. Only one significant motif was enriched in the non-canonical intron sequences.

## Supporting information

S1 Table Spontaneous genomic deletion analysis. For each predicted short intron, the sequences of the intron and flanking exons were evaluated for repetitive DNA elements.
(XLSX)

S2 Table Splicing efficiency analysis. For each cell line, splicing efficiency was calculated and is reported separately for every short intron.
(XLSX)

S3 Table Analysis of introns with the greatest read support. For the 600 short introns, with the greatest sequencing read support, we analyzed their location within transcripts as well as whether they were annotated or unannotated in existing genomic assemblies.
(XLSX)

S4 Table Non-canonical introns enriched for a sequence motif. The short non-canonical introns with the motif (from Fig 6C) are presented.
(XLSX)
